# Right ventricular function in dilated cardiomyopathy: women are the stronger sex!

**DOI:** 10.1007/s12471-015-0762-x

**Published:** 2015-10-22

**Authors:** D. Merkus

**Affiliations:** Department of Cardiology, Erasmus MC, PO Box 2040, 3000 CA Rotterdam, The Netherlands

Heart failure is mostly caused by disease of the left ventricle, but the right ventricle may play a more important role in functional capacity and prognosis of heart failure patients than is often realised. Thus, right ventricular (RV) function has been well established to be a better marker for exercise capacity than left ventricular (LV) function in both ischaemic and dilated cardiomyopathy (DCM) [1], and is correlated with clinical status and outcome in patients with ischaemic LV dysfunction [2]. Interestingly, for a comparable degree of LV dysfunction, RV function is even worse in patients with idiopathic DCM as compared with patients with ischaemic cardiomyopathy [3, 4] and, also in this patient group, is a predictor for outcome [5].

It is increasingly recognised that development, progression, prognosis and potentially also treatment of cardiovascular disease are not only dependent on the severity of disease, but are also influenced by age and gender. A potential explanation for the effect of gender on cardiovascular morbidity may be that RV function differs between men and women, with women generally having a smaller right ventricle with a higher ejection fraction (EF) [6]. Indeed, it is shown that, although the incidence of pulmonary hypertension is higher in females than males, in patients with established disease the prognosis is better in female than male patients, and that this is caused by a better preservation of RV function and a better response of the RV function to therapy, despite similar changes in the pulmonary vasculature [7].

In the present issue of the Netherlands Heart Journal, Martínez-Sellés and co-workers investigated whether differences in RV function between male and female patients could explain, at least in part, why women with DCM have a better prognosis than men. They found that RV dysfunction was only present in patients with more severe LV dysfunction (LVEF < 35) and that particularly in the group of patients with the worst LV function (LVEF < 25 %), RVEF was higher in women than men [8]. Although larger studies are required to establish causality between the effect of gender, better RV function and decreased mortality, this is a provocative study that further suggests that, also in DCM, the right ventricle plays an important role. Future studies should address whether improved RV function is correlated with improved exercise capacity, and try to identify the mechanism behind the improvement in RV function. Interestingly, in rats with pulmonary hypertension, improved outcome in female rats as compared with their male counterparts was associated with reduced RV fibrosis. As the degree of fibrosis in the left ventricle has been shown to be of predictive value for worsening of LV dysfunction in DCM [9], it would be very interesting to measure late gadolinium enhancement in magnetic resonance imaging to show a potential predictive value of RV fibrosis.

## Funding

CVON, the Netherlands Cardiovascular Research Initiative: Award Number: CVON2012-08 Phaedra.

## Conflict of interest

None declared.
